# Neurodegenerative changes in early- and late-onset cognitive impairment with and without brain amyloidosis

**DOI:** 10.1186/s13195-020-00647-w

**Published:** 2020-08-05

**Authors:** Eddie C. Stage, Diana Svaldi, Meredith Phillips, Victor Hugo Canela, Tugce Duran, Naira Goukasian, Shannon L. Risacher, Andrew J. Saykin, Liana G. Apostolova, for the Alzheimer’s Disease Neuroimaging Initiative

**Affiliations:** 1 Department of Neurology, Indiana University School of Medicine, 355 W 16th Street, Suite 4022, Indianapolis, IN 46202 USA; 2 Clinical Imaging, Eli Lilly and Company, Indianapolis, IN USA; 3 Department of Epidemiology and Biostatistics, Indiana University School of Public Health, Bloomington, IN USA; 4 Department of Biomedical Sciences Graduate School, Wake Forest University School of Medicine, Winston Salem, NC USA; 5 University of Vermont College of Medicine, Burlington, VT USA; 6 Department of Radiology and Imaging Sciences, Center for Neuroimaging, Indiana University School of Medicine, Indianapolis, IN USA; 7 Department of Medical and Molecular Genetics, Indiana University School of Medicine, Indianapolis, IN USA; 8 Indiana University Network Science Institute, Indianapolis, IN USA

**Keywords:** Early onset, Late onset, Alzheimer’s disease, AD, MRI, PET, Tau, Neurodegeneration, Hippocampal sclerosis, Limbic-predominant age-related TDP-43 encephalopathy, LATE

## Abstract

**Background:**

A substantial number of patients clinically diagnosed with Alzheimer’s disease do not harbor amyloid pathology. We analyzed the presence and extent of tau deposition and neurodegeneration in amyloid-positive (AD) and amyloid-negative (nonAD) ADNI subjects while also taking into account age of onset (< or > 65 years) as we expected that the emerging patterns could vary by age and presence or absence of brain amyloidosis.

**Methods:**

One hundred and ten early-onset AD (EOAD), 121 EOnonAD, 364 late-onset AD (LOAD), and 175 LOnonAD mild cognitive impairment (MCI) and dementia (DEM) subjects were compared to 291 ADNI amyloid-negative control subjects using voxel-wise regression in SPM12 with cluster-level family-wise error correction at *p*_FWE_ < 0.05). A subset of these subjects also received ^18^F-flortaucipir scans and allowed for analysis of global tau burden.

**Results:**

As expected, relative to LOAD, EOAD subjects showed more extensive neurodegeneration and tau deposition in AD-relevant regions. EOnonAD_MCI_ showed no significant neurodegeneration, while EOnonAD_DEM_ showed bilateral medial and lateral temporal, and temporoparietal hypometabolism. LOnonAD_MCI_ and LOnonAD_DEM_ showed diffuse brain atrophy and a fronto-temporo-parietal hypometabolic pattern. LOnonAD and EOnonAD subjects failed to show significant tau binding.

**Conclusions:**

LOnonAD subjects show a fronto-temporal neurodegenerative pattern in the absence of tau binding, which may represent underlying hippocampal sclerosis with TDP-43, also known as limbic-predominant age-related TDP-43 encephalopathy (LATE). The hypometabolic pattern observed in EOnonAD_DEM_ seems similar to the one observed in EOAD_MCI_. Further investigation into the underlying etiology of EOnonAD is warranted.

## Introduction

An estimated 5.8 million people in the USA are currently diagnosed with Alzheimer’s disease (AD) [1]. Even when rendered by dementia experts, the clinical diagnosis of AD shows only modest accuracy [2]. Twenty-nine to 56% of clinically diagnosed AD patients are AD phenocopies that fail to show AD pathology upon postmortem examination [2]. With the development of amyloid tracers for positron emission tomography (PET), we can now readily distinguish true AD cases from amyloid-negative AD phenocopies (nonAD).

Ninety-seven percent of all AD cases have symptom onset at the age of 65 or older and are classified as “late-onset” (LO), while the remaining 3% have symptom onset before the age of 65 and are termed “early-onset” (EO) [1, 3, 4]. Pathologically, patients who are younger at disease onset show greater pathological burden [5–9]. Magnetic resonance imaging (MRI), ^18^F-fluorodeoxyglucose (FDG) PET, and ^18^F-flortaucipir PET (tau PET) studies have shown that EO Alzheimer’s subjects (EOAD) have more extensive atrophy, hypometabolism, and tau burden compared to LO Alzheimer’s subjects (LOAD) [10–14]. More advanced pathologic burden in EOAD has been associated with more aggressive clinical course and is more likely to have an atypical presentation [15–18]. To our knowledge to date, the imaging biomarker profiles of early-onset nonAD (EOnonAD) have not been studied.

The Alzheimer’s Disease Neuroimaging Initiative (ADNI) is a multisite, longitudinal study that collects standardized imaging, genetic, clinical, and fluid biomarkers from clinically diagnosed amnestic mild cognitive impairment (MCI), clinically diagnosed Alzheimer’s dementia (DEM), and cognitively normal (CN) control subjects as a part of a global research effort to better understand LOAD. While the majority of ADNI subjects are older than 65 years, ADNI contains a sizeable cohort of amnestic EO MCI or DEM subjects (age of symptom onset < 65 years). The addition of amyloid imaging in the ADNI-GO/2 funding stages allowed researchers to ascertain the amyloid PET status of all ADNI participants and provided researchers the opportunity to study the biomarker-validated AD and nonAD phenocopies in greater detail.

In this study, our aim was to ascertain the extent and severity of tau and neurodegenerative pathology measured with tau PET, FDG PET, and MRI in EO and LO ADNI cohorts stratified by amyloid status as follows: EOAD MCI and DEM (EOAD_MCI_; EOAD_DEM_), EOnonAD MCI and DEM (EOnonAD_MCI_; EOnonAD_DEM_), LOAD MCI and DEM (LOAD_MCI_; LOAD_DEM_), and LOnonAD MCI and DEM (LOnonAD_MCI_; LOnonAD_DEM_). We hypothesized that EOAD and EOnonAD subjects would have more severe neurodegeneration and greater tau burden relative to their LO counterparts, indicative of the greater disease burden likely required to have equivalent impairment to the significantly older LO group. We also hypothesized that nonAD cases would have a nonAD-like pattern of neurodegeneration.

## Materials and methods

### Subjects

Data used in the preparation of this article were obtained from the ADNI database (adni.loni.usc.edu). ADNI was launched in 2003 as a public-private partnership, led by Principal Investigator Michael W. Weiner, MD. The primary goal of ADNI has been to test whether serial MRI, PET, other biological markers, and clinical and neuropsychological assessments can be combined to accurately measure and predict the progression of MCI and Alzheimer’s dementia. ADNI has undergone three complete funding cycles to date, ADNI1, ADNI-GO, and ADNI2, and is now in the ADNI3 cycle. ADNI-GO, ADNI2, and ADNI3 included ^18^F-florbetapir amyloid PET imaging.

The clinical and biomarker characteristics of the ADNI cohort have been previously published [19]. ADNI has enrolled clinically diagnosed CN, amnestic MCI, and amnestic DEM subjects. Probable AD DEM diagnosis is based on the National Institute of Neurological and Communicative Disorders and Stroke and the AD and Related Disorders Association (NINCDS-ADRDA) criteria [20]. Probable AD DEM subjects were 56 to 90 years old at enrollment, scored between 20 and 26 on the Mini-Mental State Examination (MMSE) [21] and 0.5–1 on the Clinical Dementia Rating (CDR) global score [22]. Subjects diagnosed as amnestic MCI ranged from 55 to 91 years old at enrollment, had no significant functional impairment, scored between 24 and 30 on the MMSE, had a global CDR of 0.5 (memory score ≥ 0.5), and had impairment on Wechsler Memory Scale – Logical Memory II test [23]. CN subjects had MMSE between 24 and 30 and a global CDR of 0 and did not meet criteria for MCI or DEM. Subjects were excluded due to inability to undergo MRI or if they had other neurological disorders, active depression, or history of psychiatric diagnosis, alcohol, or substance dependence within the past 2 years, less than 6 years of education or were not fluent in English or Spanish. The full list of inclusion/exclusion criteria may be accessed in the online ADNI protocol (http://www.adni-info.org/Scientists/ADNIStudyProcedures.html). Written informed consent was obtained from all participants, and the institutional review board (IRB) at all ADNI sites have reviewed and approved ADNI data collection protocol.

For our analysis, we used 231 EO subjects with reported age of symptom onset ≤ 65 years from the ADNI database with available ^18^F-florbetapir amyloid PET or CSF Aβ data (219 of the 231 received ^18^F-Florbetapir PET, while the remaining 12 had CSF Aβ data). One hundred seventythree EO subjects met criteria for MCI and 58 for DEM. Sixty MCI and 50 DEM were amyloid-positive (EOAD_MCI_ and EOAD_DEM_), and 113 and 8, respectively, were amyloid-negative (EOnonAD_MCI_ and EOnonAD_DEM_) based on previously validated ^18^F-florbetapir global means standard uptake volume ratio (SUVR) cut-off of 1.17 [24] or a CSF Aβ_1–42_ level < 192 pg/ml [25]. The subset with tau PET (^18^F-Flortaucipir) included 10 EOAD_MCI_, 7 EOAD_DEM_, 38 EOnonAD_MCI_, and 3 EOnonAD_DEM_.

LO subjects included had either ^18^F-florbetapir amyloid PET (488/539 subjects) or CSF Aβ data (51/539 subjects), and all had FDG PET and MR imaging. Special care was taken to ensure that no LO diagnostic group was significantly more or less cognitively impaired than their EO counterpart (measured by MMSE) by removing outlier subjects, resulting in 539 subjects. Three hundred sixty-seven met criteria for MCI and 172 for DEM. Two hundred sixteen LO MCI and 148 LO DEM were amyloid-positive (LOAD_MCI_ and LOAD_DEM_), while 151 LO MCI and 24 LO DEM were amyloid-negative (LOnonAD_MCI_; LOnonAD_DEM_). The subset with tau PET included 53 LOAD_MCI_, 27 LOAD_DEM,_ 51 LOnonAD_MCI_, and 2 LOnonAD_DEM._ Due to the low numbers of EO and LO nonAD_DEM_ subjects with ^18^F-flortaucipir scans (3 and 2, respectively), they were grouped together with the EO and LOnonAD_MCI_, resulting in 41 EOnonAD and 53 LOnonAD subjects in the tau comparisons.

Since EO and LO groups cannot be directly compared because neurodegenerative changes associated with aging could inadvertently confound the results, we conducted two sets of analyses. We first compared each EO and LO group to the same CN comparison group comprised of the 291 amyloid-negative (SUVR< 1.17) CN subjects within the age range of 55 to 90 years old. This comparison allowed for a straightforward interpretation of the effect sizes as a measure of disease impact. Next, we repeated the analyses comparing the LO and EO groups to only the older (*N* = 146) and younger half (*N* = 145) of CN, respectively. The latter results are presented in Additional figures 1, 2, 3, and 4 and Additional tables 1 & 2.

### MRI and PET acquisition and analyses

ADNI MRI and PET acquisition and preprocessing protocols can be found at www.adni-info.org. The MRI data acquisition and preprocessing have been previously described elsewhere [20]. We downloaded preprocessed MRI data from LONI IDA (https://ida.loni.usc.edu). Scans were spatially warped to Montreal Neurological Institute (MNI) space and segmented into gray matter (GM), white matter, and CSF components using voxel-based morphometry (VBM) in Statistical Parametric Mapping version 12 (SPM12), as described previously [26]. GM maps were normalized and smoothed using 10-mm full-width half maximum (FWHM) Gaussian kernel, which yielded gray matter density (GMD) data. Intracranial volume (ICV) was also calculated using FreeSurfer version 5.1.

PET scanners across sites were held to rigorous qualifications, calibration, and normalization standards as described in detail elsewhere [22]. We downloaded preprocessed amyloid, FDG PET, and tau PET data from LONI IDA (https://ida.loni.usc.edu). The scans were already averaged, aligned to standard space, re-sampled to a standard image and voxel size (2 mm × 2 mm × 2 mm), and smoothed to a uniform resolution as previously described [27]. We aligned the images to the corresponding MRI scan from the same visit and normalized them to MNI space using parameters obtained from the MRI segmentation using SPM12. PET scans were intensity normalized to mean pons uptake for FDG, whole cerebellum for amyloid and cerebellar crus for tau PET, resulting in whole brain SUVR images as previously described [28, 29]. To assign subjects into groups by amyloid status, we used an amyloid PET cutoff of SUVR ≥ 1.17 or CSF Aβ_1–42_ level < 192 pg/ml [24, 25].

### Statistical analyses

#### Clinical and demographic analyses

The statistical distribution of clinical and demographic characteristics (age, education, global CDR, MMSE, and amyloid PET mean global SUVR) were analyzed in SPSS version 24.2 using one-way ANOVA. ANOVA *p* values are listed in the tables and Bonferroni-corrected multiple comparisons *p* values are listed in the results text. *APOE4* genotype and sex frequency comparisons were done using a chi-square test with two-sided *p* values. The alpha value for all comparisons was *p* < 0.05. In addition, a mixed effects model was generated in SAS 9.4 to comparing change in cortical [^18^F]-florbetapir SUVR to the CN group as a reference.

#### Parametric mapping

We used voxel-wise linear regression models in SPM12 to study the extent and severity of neurodegeneration and tau burden in EO and LO AD and nonAD groups relative to CN while controlling for age, sex, and education. Additionally, in the MRI analyses, we covaried for MRI field strength (1.5 T vs. 3 T) and ICV. Family-wise error (FWE) cluster-level correction was applied to correct for multiple comparisons with a significance threshold of *p* < 0.01. Because side-by-side interpretation of significance maps generated with unequal sample sizes can be misleading, we also derived β-coefficient maps to demonstrate the effect sizes in each comparison, which are visualized using the MRIcrogl version 2.1 software.

## Results

### AD analyses

#### Demographic comparisons

The demographic, neuropsychological, and amyloid burden comparisons of the amyloid positive diagnostic groups relative to CN are shown in Table 1. Our CN were significantly older than the EOAD_MCI_ and EOAD_DEM_ and significantly younger than the LOAD_MCI_ and LOAD_DEM_ subjects (*p* < 0.001, both). The CN subjects were significantly less impaired (global CDR and MMSE), had significantly fewer *APOE4* carriers, and had a significantly lower [^18^F]-florbetapir SUVR compared to all cognitively impaired AD groups (*p* < 0.001, all). In addition, EOAD_DEM_ and both LOAD_MCI_ and LOAD_DEM_ had significantly fewer years of education than the CN group (*p* = 0.006, *p* < 0.001, and *p* < 0.001, resp). When the data were split into younger and older subgroups, the effects remained the same, except that LOAD_MCI_ subjects were significantly younger (*p* < 0.001) and LOAD_DEM_ no longer were significantly older or younger than the CN group (Additional Table 1).

**Table 1:** EOAD and LOAD demographic comparisons to CN. The comparisons were done using ANOVA and chi-square tests with two-sided *p* values. The Bonferroni-corrected pairwise differences *relative to CN* are discussed in the “Results” section. Significant *p* values (< 0.05) are bolded

Variable	CN (***N*** = 291)	EOAD_**MCI**_ (***N*** = 60)	EOAD_**DEM**_ (***N*** = 50)	***p*** value	LOAD_**MCI**_ (***N*** = 216)	LOAD_**DEM**_ (***N*** = 148)	***p*** value
**Age, years, mean (SD)**	74.3 (6.4)	65.4 (6.0)	64.7 (6.3)	**< 0.001**	76.4 (5.8)**	78.3 (5.9)**	**< 0.001**
**Sex, male %**	52.2	46.7	44.0	0.461	60.2	58.8	0.174
**Education, years, mean (SD)**	16.7 (2.6)	16.7 (2.8)*	15.6 (2.4)*	**0.022**	15.8 (2.8)	15.4 (3.0)	**< 0.001**
**%*APOE*ε4, 0/1/2 alleles**	77/22/1	18/52/30	26/38/36	**< 0.001**	34/52/14	26/56/18	**< 0.001**
**Global CDR, mean (SD)**	0.02 (0.09)	0.50 (0.00)***	0.87 (0.33)***	**< 0.001**	0.50 (0.16)***	0.84 (0.36)***	**< 0.001**
**MMSE, mean (SD)**	29.0 (1.3)	27.8 (1.8)***	22.5 (3.3)***	**< 0.001**	27.4 (1.9)***	23.0 (2.8)***	**< 0.001**
**Global cortical [**^**18**^**F]-florbetapir SUVR, mean (SD)**	1.03 (0.06)	1.41 (0.15)*	1.48 (0.13)*	**< 0.001**	1.43 (0.17)*	1.47 (0.16)*	**< 0.001**
**Change in cortical [**^**18**^**F]-florbetapir SUVR referenced to CN (*p*value)**	ref	0.0155 (0.0352)	0.0079 (0.5071)	N/A	0.0078 (0.1449)	0.0003 (0.9692)	N/A
**Tau scans,*N***	126	10	7	N/A	53	27	N/A

*MCI and DEM significantly different at *p* < 0.05

**MCI and DEM significantly different at *p* < 0.01

***MCI and DEM significantly different at *p* < 0.001

As expected, the EOAD_MCI_ and EOAD_DEM_ subjects were significantly younger compared to the LOAD_MCI_ and LOAD_DEM_ groups (*p* < 0.001, Table 2, top). Compared to EOAD_MCI_, LOAD_MCI_ had significantly fewer years of education (*p* = 0.021). The EOAD_DEM_ and LOAD_DEM_ groups showed similar education. Both EOAD groups had a significantly higher percentage of *APOE ε4* homozygotes compared to LOAD subjects (MCI *p* = 0.004, DEM *p* = 0.022). There were no significant differences in global ^18^F-florbetapir SUVR, global CDR, or MMSE between EO and LOAD groups.

**Table 2:** EO vs. LO demographic comparisons. The comparisons were done using ANOVA and chi-square tests with two-sided *p* values. Significant *p* values (< 0.05) are bolded

**Variable**	**EOAD**_**MCI**_**(*N*** **= 60)**	**LOAD**_**MCI**_**(*N*** **= 216)**	***p*value**	**EOnonAD**_**MCI**_**(*N*** **= 113)**	**LOnonAD**_**MCI**_**(*N*** **= 151)**	***p*value**
**Age, years, mean (SD)**	65.4 (6.0)	76.4 (5.8)	**< 0.001**	65.5 (5.8)	77.6 (6.2)	**< 0.001**
**Sex, male %**	46.7	60.2	0.065	50.4	59.6	0.138
**Education, years, mean (SD)**	16.7 (2.8)	15.8 (2.8)	**0.021**	16.2 (2.5)	16.3 (2.5)	0.848
**%*APOE e4*, 0/1/2 alleles**	18/52/30	34/52/14	**0.004**	68/30/2	86/13/2	**0.005**
**Global CDR, mean (SD)**	0.50 (0.00)	0.50 (0.16)	0.823	0.46 (0.17)	0.48 (0.11)	0.167
**MMSE, mean (SD)**	27.8 (1.8)	27.4 (1.9)	0.229	28.6 (1.5)	28.4 (1.6)	0.175
**Global cortical [**^**18**^**F]-florbetapir SUVR, mean (SD)**	1.41 (0.15)	1.43 (0.17)	0.315	1.03 (0.08)	1.01 (0.09)	0.115
**Tau scans,*N***	10	53	N/A	38	51	N/A
**Diagnostic group (*N*)**	**EOAD**_**DEM**_**(*N*** **= 50)**	**LOAD**_**DEM**_**(*N*** **= 148)**	***p*value**	**EOnonAD**_**DEM**_**(*N*** **= 8)**	**LOnonAD**_**DEM**_**(*N*** **= 24)**	***p*value**
**Age, years, mean (SD)**	64.7 (6.3)	78.3 (5.9)	**< 0.001**	66.3 (5.8)	79.4 (5.8)	**< 0.001**
**Sex, male %**	44.0	58.8	0.069	50.0	83.3	0.059
**Education, years, mean (SD)**	15.6 (2.4)	15.4 (3.0)	0.668	15.6 (3.5)	15.6 (3.0)	1.000
**%*APOE e4*, 0/1/2 alleles**	26/38/36	26/56/18	**0.022**	71/14/14	83/13/4	0.642
**Global CDR, mean (SD)**	0.87 (0.33)	0.84 (0.36)	0.579	0.69 (0.26)	0.83 (0.24)	0.155
**MMSE, mean (SD)**	22.5 (3.3)	23.0 (2.8)	0.283	23.0 (2.4)	23.6 (1.9)	0.453
**Global cortical [**^**18**^**F]-florbetapir SUVR, mean (SD)**	1.48 (0.13)	1.47 (0.16)	0.616	1.04 (0.08)	1.01 (0.10)	0.485
**Tau scans,*N***	7	27	N/A	3	2	N/A

Regional amyloid comparisons between the AD subjects showed a significant difference (*p* = 0.044) in parietal cortices and significant (*p* = 0.048) difference in temporal amyloid SUVR between EOAD_MCI_ and EOAD_DEM_ (Additional Table 2).

#### Imaging comparisons

**Fig. 1:**
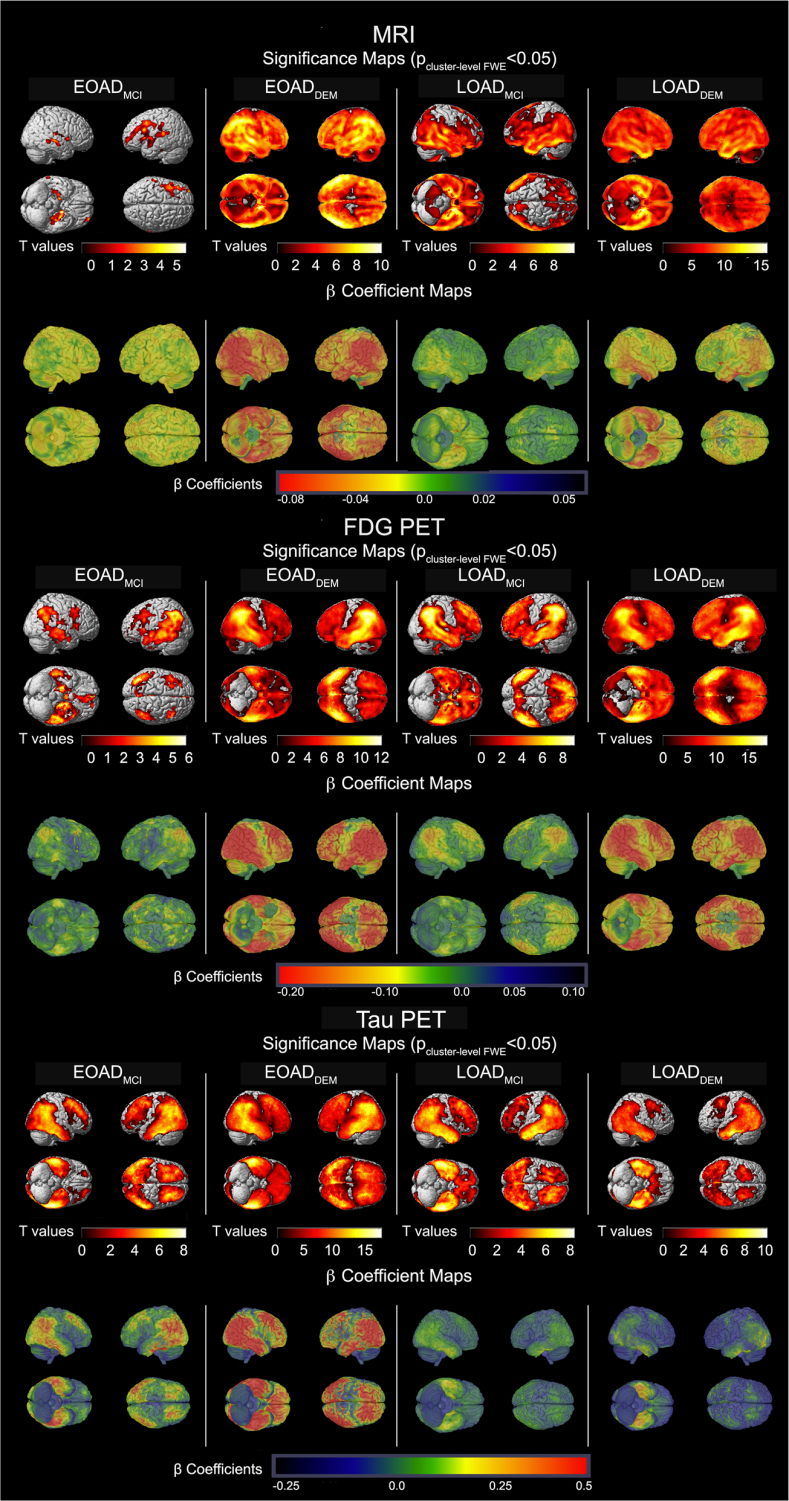
MRI (top), FDG PET (middle), and tau PET (bottom) comparisons between the AD groups and CN. The significance maps show *p* < 0.05 thresholded FWE cluster-level corrected results of EOAD_MCI_ (*N* = 60), EOAD_DEM_ (*N* = 50), LOAD_MCI_ (*N* = 216), and LOAD_DEM_ (*N* = 148) vs. CN (*N* = 291). The results displayed here are for all subjects with available scans in each modality

The FWE cluster-level corrected maps of the MRI, FDG PET, and tau PET comparisons of EOAD and LOAD spectrum individuals to CN are shown in Fig. 1. The same analyses limited to only subjects with tau PET scans are shown in Additional Figure 1 while Additional Figure 2 displays comparisons of EOAD and LOAD to younger and older CN subgroups, respectively. The pattern of neurodegeneration and tau deposition seen in Fig. 1 and Additional Figures 1 and 2 are very similar discounting the probability of exaggerated age or selection bias.

##### *MRI* (Fig. 1, top panel)

The EOAD_MCI_ group showed two significant clusters of atrophy in the left and right medial and lateral temporal and left frontal cortices relative to the CN group (left cluster: cluster size *k* = 45,797, cluster *p*_FWE_ < 0.001; right cluster: *k* = 20,760, cluster *p*_FWE_ = 0.003). Compared to the CN group, the LOAD_MCI_ cohort showed significant atrophy of the bilateral medial and lateral temporal, temporoparietal, insular, occipital, and frontal regions (single cluster *k* = 688,646, cluster *p*_FWE_ < 0.001). The EOAD_MCI_ group visually showed a larger effect size (i.e., more severe atrophy) than LOAD_MCI_ in overlapping regions (see β-coefficient maps in Fig. 1, top panel).

Both EOAD_DEM_ and LOAD_DEM_ showed extensive atrophy throughout the brain compared to CN (single clusters, *k*_EO_ = 1,541,575, *k*_LO_ = 1,503,763, cluster *p*_FWE_ < 0.001 for both). The significance and β-coefficient maps show a stronger effect size (i.e., more severe atrophy) in the EOAD_DEM_ than the LOAD_DEM_ group (see β-coefficient maps in Fig. 1, top panel).

##### *FDG PET* (Fig. 1, middle panel)

Compared to CN, EOAD_MCI_ showed a significant hypometabolic cluster in bilateral medial and lateral temporal and lateral, temporoparietal cortices (*k* = 32,246, cluster *p*_FWE_ < 0.001). Additionally, there was a small cluster of hypometabolism in bilateral dorsolateral prefrontal cortex (*k* = 3396, cluster *p*_FWE_ < 0.001). LOAD_MCI_ subjects showed hypometabolism of the bilateral inferior temporal, medial and lateral temporal, and parietal cortices as well as bilateral frontal cortex (single cluster, *k* = 94,307, cluster *p*_FWE_ < 0.001, Fig. 1, middle panel).

Both the EOAD_DEM_ and LOAD_DEM_ groups showed extensive hypometabolism relative to CN in bilateral parietal, temporal, and frontal lobes, as well as insular and cingulate cortices (single clusters, *k*_EO_ = 148,701, *k*_LO_ = 185,998, cluster *p*_FWE_ < 0.001 for both). As with the MRI analysis, the EOAD_DEM_ group showed a stronger effect size (i.e., more severe hypometabolism) than the LOAD_DEM_ group (see β-coefficient maps in Fig. 1, middle panel).

##### *Tau PET* (Fig. 1, bottom panel)

These analyses were limited to the subset of individuals with available tau PET imaging. Compared to the CN group, the EOAD_MCI_ group had a significant cluster of tau binding covering temporal, parietal, parietooccipital, and right frontal cortices (*k* = 74,981, cluster *p*_FWE_ < 0.001). An additional significant cluster of tau binding was present in the left prefrontal cortex (*k* = 9800, cluster *p*_FWE_ < 0.001). The LOAD_MCI_ cohort showed tau deposition in a similar pattern (single cluster, *k* = 96,885, cluster *p*_FWE_ < 0.001). The beta coefficient maps demonstrated greater tau burden in EOAD_MCI_ compared to LOAD_MCI_ (see β-coefficient maps in Fig. 1, bottom panel).

EOAD_DEM_ showed tau binding in all cortical regions save for the primary sensorimotor and visual cortices (single cluster, *k* = 157,966, cluster *p*_FWE_ < 0.001). LOAD_DEM_ showed two significant clusters of tau binding—one in the posterior association cortices (*k* = 67,260, cluster *p*_FWE_ < 0.001) and a smaller one in the bilateral prefrontal cortices (*k* = 4931, cluster *p*_FWE_ < 0.001). The β-coefficient maps show much more severe and extensive tau deposition in EOAD_DEM_ compared to LOAD_DEM_ (see β-coefficient maps in Fig. 1, bottom panel).

### NonAD analyses

#### Demographic comparisons

Direct comparisons of CN to EOnonAD_MCI_, LOnonAD_MCI,_ EOnonAD_DEM_, and LOnonAD_DEM_ showed the expected significant difference in age, global CDR, and MMSE (*p* < 0.001, Table 3). Compared to CN, LOnonAD_DEM_ had a greater proportion of men (*p* = 0.003) and lower education (*p* = 0.044). Even when split in younger and older subgroups, the age differences between CN and the respective disease groups remained significant with the exception of LOnonAD_DEM_ (Additional Table 3).

**Table 3:** EOnonAD and LOnonAD demographic comparisons to CN. The comparisons were done using ANOVA and chi-square tests with two-sided *p* values. The Bonferroni-corrected pairwise differences *relative to CN* are discussed in the “Results” section. Significant *p* values (< 0.05) are bolded

Variable	CN (***N*** = 291)	EOnonAD_**MCI**_ (***N*** = 113)	EOnonAD_**DEM**_ (***N*** = 8)	***p*** value	LOnonAD_**MCI**_ (***N*** = 151)	LOnonAD_**DEM**_(***N*** = 24)	***p*** value
**Age, years, mean (SD)**	74.3 (6.4)	65.5 (5.8)	66.3 (5.8)	**< 0.001**	77.6 (6.2)	79.4 (5.8)	**< 0.001**
**Sex, male %**	52.2	50.4	50.0	0.944	59.6*	83.3*	**0.008**
**Education, years, mean (SD)**	16.7 (2.6)	16.2 (2.5)	15.6 (3.5)	0.095	16.3 (2.5)	15.6 (3.0)	**0.037**
**%*APOE*ε4, 0/1/2 alleles**	77/22/1	68/30/2	71/14/14	**0.028**	86/13/2	83/13/4	0.173
**Global CDR, mean (SD)**	0.02 (0.09)	0.46 (0.17)***	0.69 (0.26)***	**< 0.001**	0.48 (0.11)***	0.83 (0.24)***	**< 0.001**
**MMSE, mean (SD)**	29.0 (1.3)	28.6 (1.5)***	23.0 (2.4)***	**< 0.001**	28.4 (1.6)***	23.6 (1.9)***	**< 0.001**
**Global cortical [**^**18**^**F]-florbetapir SUVR, mean (SD)**	1.03 (0.06)	1.03 (0.08)	1.04 (0.08)	0.214	1.01 (0.09)	1.01 (0.10)	0.160
**Change in cortical [**^**18**^**F]-florbetapir SUVR referenced to CN (*p*value)**	ref	− 0.0049 (0.1835)*	− 0.0387 (**0.0175**)*	N/A	− 0.0099 (**0.0091**)	0.0093 (0.5425)	N/A
**Tau scans,*N***	126	38	3	N/A	51	2	N/A

*MCI and DEM significantly different at *p* < 0.05

***MCI and DEM significantly different at *p* < 0.001

By definition, EOnonAD_MCI_ and EOnonAD_DEM_ were significantly younger than the corresponding LOnonAD groups (*p* < 0.001). The EOnonAD_MCI_ group had a higher proportion of *APOE ε4* carriers compared to the LOnonAD_MCI_ group (*p* = 0.005). There were no significant differences in sex, education, global CDR, MMSE, or global ^18^F-florbetapir SUVR between the groups.

A closer look into regional amyloid differences among amyloid-negative subjects revealed significant differences between the LOnonAD_DEM_ group and CN, EOnonAD_MCI_, EOnonAD_DEM_, and LOnonAD_MCI_ in parietal SUVR (*p* < 0.05 for all). No other region was significantly different.

#### Imaging comparisons

**Fig. 2:**
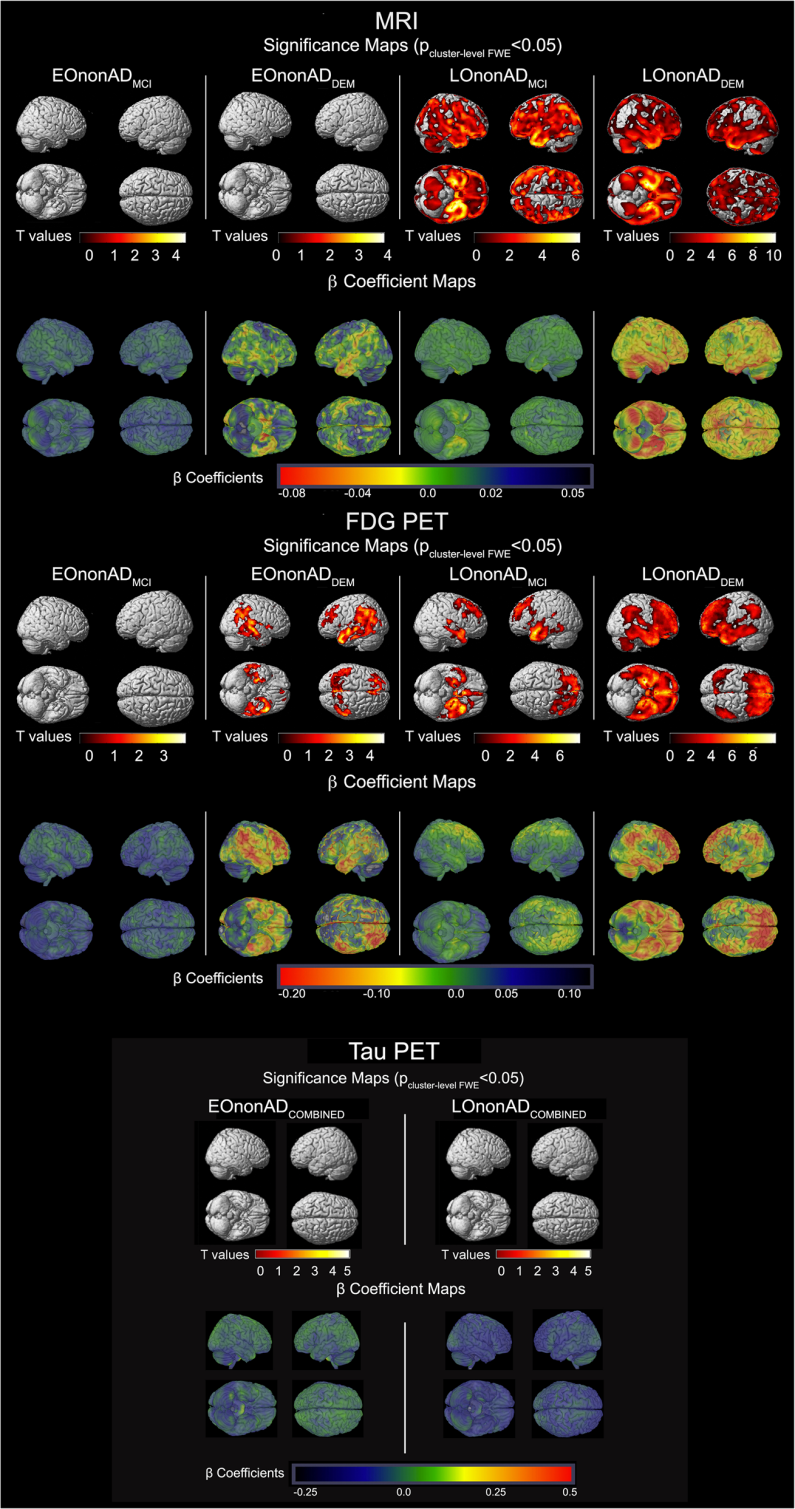
MRI (top), FDG PET (middle), and tau PET (bottom) comparisons between the nonAD groups and CN. The significance maps show *p* < 0.05 thresholded FWE cluster-level corrected results of EOnonAD_MCI_ (*N* = 113), EOnonAD_DEM_ (*N* = 8), LOnonAD_MCI_ (*N* = 151), and LOnonAD_DEM_ (*N* = 24) vs. CN (*N* = 291). The results displayed here are for all subjects with available scans in each modality

The FWE cluster-level corrected MRI, FDG PET, and tau PET comparison maps of the nonAD groups to CN are shown in Fig. 2. The same analyses limited to only subjects with tau PET scans are shown in Additional Figure 3, while Additional Figure 4 displays comparisons of EOnonAD and LOnonAD to younger and older CN subgroups, resp. The pattern of neurodegeneration and tau deposition seen in Additional Figure 4 is largely identical to the one in Fig. 2, with the exception of emerging tau deposition in bilateral frontal and right parietal lobes in EOnonAD when compared to the young CN.

##### *MRI* (Fig. 2, top panel)

EOnonAD showed no significant atrophy compared to the CN group. LOnonAD_MCI_ showed extensive atrophy in the bilateral medial and lateral temporal, temporoparietal, parietooccipital, and frontal cortices (single cluster, *k* = 569,219, cluster *p*_FWE_ < 0.001). LOnonAD_DEM_ had similarly widespread atrophy showing two significant clusters—one in bilateral temporoparietal and frontal cortices (*k* = 602,716, cluster *p*_FWE_ < 0.001) and another in the cerebellum (*k* = 13,494, cluster *p*_FWE_ = 0.020). The largest effect size was observed in LOnonAD_DEM_ with greatest predilection for the medial and inferior temporal lobes (see β-coefficient maps in Fig. 2, top panel).

##### *FDG PET* (Fig. 2, middle panel)

Compared to CN, EOnonAD_MCI_ showed no significant hypometabolism, while the EOnonAD_DEM_ group showed three significant clusters in left and right temporoparietal (left: *k* = 15,114, cluster *p*_FWE_ < 0.001; right: *k* = 6104, cluster *p*_FWE_ < 0.001) and bilateral frontal cortices (single cluster, *k* = 3002, cluster *p*_FWE_ < 0.001). LOnonAD_MCI_ showed a significant cluster of hypometabolism in bilateral temporal and prefrontal cortices (*k* = 24,278, cluster *p*_FWE_ < 0.001). A similar fronto-temporal pattern of hypometabolism was also observed in LOnonAD_DEM;_ however, it also extended to the parietal lobes (single cluster, *k* = 78,550, cluster *p*_FWE_ < 0.001).

##### *Tau PET* (Fig. 2, bottom panel)

Due to the small sample sizes, the EOnonAD and LOnonAD groups were not split by disease stage. There was no significant tau binding in either EOnonAD or LOnonAD. As mentioned above, EOnonAD showed tau deposition in bilateral frontal (single cluster, *k* = 7215, cluster *p*_FWE_ < 0.001) and right parietal lobes (single cluster, *k* = 4664, cluster *p*_FWE_ < 0.001) when compared to young CN only (Additional Figure 4).

## Discussion

The current study aimed to map neurofibrillary, structural, and metabolic differences between EO and LO MCI and DEM subjects stratified by amyloid positivity. As expected, we found that EOAD_MCI_ and EOAD_DEM_ subjects show more severe neurodegeneration and greater tau deposition compared to LOAD_MCI_ and LOAD_DEM_, respectively, a finding that is consistent with previous imaging reports [5–11, 14, 18], and with the fact that EO individuals have a much more aggressive disease course [15, 18].

The availability of amyloid PET imaging or CSF Aβ measurements allowed us to identify nonAD cases that were enrolled as Alzheimer’s phenocopies. While we failed to find significant neurodegeneration in EOnonAD_MCI_, we observed significant hypometabolism in EOnonAD_DEM_ in the absence of significant atrophy, a finding that could be indicative of synaptic dysfunction before cellular loss. The lack of findings, particularly in the EOnonAD_MCI_ subjects, where the neurodegenerative changes are likely subtle, may be due to our inability to properly account for age-related degeneration (despite covarying for age during the analysis) when comparing directly to the CN subjects who are significantly older. In our EOnonAD_DEM_ subjects, the lack of significant atrophy may be due to a relatively small sample size, as the beta-coefficient maps indicate a pattern of neurodegeneration similar to that seen in the FDG PET analysis. Furthermore, the EOnonAD_DEM_ subjects may be a heterogeneous group of multiple etiologies, making detection of significant clusters of atrophy difficult.

LOnonAD cases showed pronounced atrophy and hypometabolism with greatest predilection for the temporal and frontal lobes. This pattern of neurodegeneration has been reported in primary age-related tauopathy (PART) and hippocampal sclerosis with TAR-DNA binding protein 43 (TDP-43) inclusions (HS-TDP-43) [30, 31]. Both conditions are highly prevalent among the elderly with and without cognitive deficit [30, 31]; however, of these two, HS-TDP-43 (also known as limbic-predominant age-related TDP-43 encephalopathy (LATE) [32]) is the more likely etiology due to the lack of tau binding in the medial temporal lobes which is expected in PART. It is worth noting that though ^18^F-flortaucipir binds well to mature tangles in 3R+4R tauopathies, such as in AD and PART [33, 34], further post mortem studies are needed to say with confidence which tauopathy tau variants can reliably be bound with flortaucipir.

The hypometabolic pattern we observed in LOnonAD fits well with previous pathologic and imaging reports of LATE. TDP-43 inclusions and neurite deposits first appear in the hippocampal dentate granule cells, subiculum, and the amygdala [31, 35, 36]. In more advanced stages, TDP-43 pathology is also found in frontal and temporal neocortex [31, 35, 36]. TDP-43 pathology is extremely prevalent among cognitively impaired elderly and is the stand-alone pathology in 4.2% of these cases [31, 35, 37, 38]. Eighty-six percent of TDP-43-positive cases have HS-TDP-43 [31, 39–41]. HS-TDP-43 oftentimes show episodic and semantic memory dysfunction [42], explaining how they could easily be diagnosed clinically with AD. Individuals with HS-TDP-43 have greater hippocampal atrophy and greater cognitive impairment than those with HS without TDP-43 [35, 36, 39]. Additional support for our hypothesis that our LOnonAD subjects likely harbor HS-TDP-43 are the recent reports that HS-TDP-43 cases show hypometabolic changes in the medial and lateral temporal, posterior and middle cingulate, precuneus, and prefrontal cortex [43], similar to the FDG PET pattern we observed in LOnonAD_MCI_ and LOnonAD_AD_. Similar hypometabolic and atrophy patterns involving medial and lateral temporal and prefrontal cortices were recently reported in two additional clinic-pathologic studies [44, 45].

An additional possibility is that some LOnonAD subjects may suffer from behavioral variant frontotemporal dementia (bvFTD). However, this is less likely given the mean age of our LOnonAD cohorts (77.6 and 79.4 years, resp) and their amnestic predominant presentation at enrollment as required by ADNI (see http://www.adni-info.org/Scientists/ADNIStudyProcedures.html). It is worth noting, however, that in rare cases (10% of pathologically confirmed bvFTD cases), patients presented with primarily amnestic symptoms and some studies have even reported as much as 25% of pathologically confirmed FTLD cases to have a disease onset after the age of 65 [46, 47].

Several strengths and limitations of our study should be noted. One of the strengths is the relatively large sample size of EO subjects available through ADNI. Additionally, ADNI employs meticulously standardized clinical and imaging data collection, which is routinely subjected to quality control. One of the limitations of our analyses is the cross-sectional design and the measurement of atrophy, which has a temporal component. This means that we are actually measuring differences in gray matter density, which implies atrophy, but is not synonymous. Longitudinal analyses are needed to assess atrophy and metabolic changes over time. Additionally, while the rigorous exclusion criteria employed in ADNI are typical of clinical trials, this renders the ADNI’s population as not representative of the general population. Furthermore, there is very little post mortem data currently available for ADNI, which means diagnosis of AD largely lacks pathological verification. Finally, while we are including the EOnonAD_DEM_ in our report for completeness, one must keep in mind that the number of subjects in this group is very small; thus, the findings should be interpreted with caution. Larger research studies such as the recently funded Longitudinal Early-onset Alzheimer’s disease Study (LEADS) which will use amyloid imaging and detect EOnonAD cases will be able to define the neurodegenerative pattern in this group.

## Conclusion

In conclusion, our study found a similar neurodegenerative pattern between amnestic amyloid-positive EO and LO MCI and DEM subjects. These processes were more severe in the EO group indicating a more aggressive disease course. We also found that LOnonAD_DEM_ subjects show anterior temporal neurodegeneration which might reflect the presence HS-TDP-43 or LATE. In the absence of reliable in vivo TDP-43 biomarker, the only feasible method of confirmation is through post-mortem examination of the brains. Other large research consortia such as the recently funded LEADS project will allow us the opportunity to systematically study EOAD and EOnonAD and further characterize these highly understudied disease states.

## Supplementary information

**Additional Table 1.** EOAD and LOAD demographic comparisons to the young and old CN groups, resp. The comparisons were done using ANOVA and chi-square tests with two-sided *p*-values. The Bonferroni-corrected pairwise differences **relative to CN** are discussed in the Results section. Significant *p*-values (< 0.05) are bolded.

**Additional Table 2.** Regional amyloid PET (18F-Florbetapir) comparisons between amyloid positive subjects for frontal, cingulate, parietal and temporal cortices.

**Additional Table 3.** EOnonAD and LOnonAD demographic comparisons to the young and old CN groups, resp. The comparisons were done using ANOVA and chi-square tests with two-sided *p*-values. The Bonferroni-corrected pairwise differences **relative to CN** are discussed in the Results section. Significant *p*-values (< 0.05) are bolded.

**Additional Table 4.** Regional amyloid PET (18F-Florbetapir) comparisons between amyloid negative subjects for frontal, cingulate, parietal and temporal cortices.

**Additional Figure 1.** MRI (top), FDG PET (middle), tau PET (bottom) comparisons between the AD and CN groups restricted to only subjects with available tau PET scans. The significance maps show *p* < 0.05 thresholded FWE cluster-level corrected results. of EOAD_MCI_ (*N* = 10), EOAD_DEM_ (*N* = 7), LOAD_MCI_ (*N* = 53) and LOAD_DEM_ (*N* = 27) vs. CN (*N* = 126).

**Additional Figure 2.** MRI (top), FDG PET (middle), tau PET (bottom) comparisons between young CN and EOAD and old CN and LOAD groups. The significance maps show p < 0.05 thresholded FWE cluster-level corrected results of EOAD_MCI_ (*N* = 60) and EOAD_DEM_ (*N* = 50) vs young CN (*N* = 145), LOAD_MCI_ (*N* = 216) and LOAD_DEM_ (*N* = 148) vs. old CN (*N* = 146).

**Additional Figure 3.** MRI (top), FDG PET (middle), tau PET (bottom) comparisons between the nonAD and CN groups restricted to only subjects with available tau PET scans. The significance maps show p < 0.05 thresholded FWE cluster-level corrected results of EOnonAD_MCI_ (*N* = 38), EOnonAD_DEM_ (*N* = 3), LOnonAD_MCI_ (*N* = 51) and LOnonAD_DEM_ (*N* = 2) vs. CN (*N* = 126).

**Additional Figure 4.** MRI (top), FDG PET (middle), tau PET (bottom) comparisons between young CN and EOnonAD and old CN and LOnonAD groups. The significance maps show p < 0.05 thresholded FWE cluster-level corrected results of EOnonAD_MCI_ (*N* = 113) and EOnonAD_DEM_ (*N* = 8) vs young CN (*N* = 145), LOnonAD_MCI_ (*N* = 151) and LOnonAD_DEM_ (*N* = 24) vs. old CN (*N* = 146).

## Data Availability

All data presented is available through the ADNI website at http://adni.loni.usc.edu/data-samples/access-data/. Investigators may apply for access by filling out the online application form and adhering to the data use agreement.

## Abbreviations

AD: Alzheimer’s disease
ADNI: Alzheimer’s Disease Neuroimaging Initiative
CDR: Clinical dementia rating
CN: Cognitively normal
DEM: Dementia
EO: Early onset
EOAD: Early onset Alzheimer’s disease
FDG: Fluorodeoxyglucose
FWE: Family-wise error
FWHM: Full-width half-max
GMD: Gray matter density
HS: Hippocampal sclerosis
ICV: Intracranial volume
LO: Late onset
LOAD: Late onset Alzheimer’s disease
LATE: Limbic-predominant age-related TDP-43 encephalopathy
MCI: Mild cognitive impairment
MMSE: Mini-mental state exam
MNI: Montreal Neurological Institute
PART: Primary age-related tauopathy
nonAD: Amyloid negative
SUVR: Standardized uptake value ratio
TDP-43: TAR-DNA binding protein 43
