# Supplementary material for: Neurodegenerative changes in early- and late-onset cognitive impairment with and without brain amyloidosis
Source: Alzheimers Res Ther. 2020 Aug 5;12:93. doi: 10.1186/s13195-020-00647-w (PMC7409508; doi:10.1186/s13195-020-00647-w)
Supplement: Supplementary file 1 — Additional Table 1. EOAD and LOAD demographic comparisons to the young and old CN groups, resp. The comparisons were done using ANOVA and chi-square tests with two-sided p-values. The Bonferroni-corrected pairwise differences relative to CN are discussed in the Results section. Significant p-values (< 0.05) are bolded. [file 13195_2020_647_MOESM1_ESM.docx]

| **Variable** | **Young CN**  **(N=145)** | **EOAD_MCI_ (N=60)** | **EOAD_DEM_**  **(N=50)** | **p-value** | **Old CN**  **(N=146)** | **LOAD_MCI_**  **(N=216)** | **LOAD_DEM_ (N=148)** | **p-value** |
| --- | --- | --- | --- | --- | --- | --- | --- | --- |
| **Age, years, Mean (SD)** | 69.1 (3.3) | 65.4 (6.0)*** | 64.7 (6.3)*** | **<0.001** | 79.4 (4.3) | 76.4 (5.8)*** | 78.3 (5.9) | **<0.001** |
| **Sex,**  **Male %** | 50.3 | 46.7 | 44.0 | 0.713 | 54.1 | 60.2 | 58.8 | 0.505 |
| **Education, years, Mean (SD)** | 16.7 (2.5) | 16.7 (2.8) | 15.6 (2.4)** | **0.027** | 16.7 (2.7) | 15.8 (2.8)** | 15.4 (3.0)*** | **<0.001** |
| **% *APOE* ε4, 0/1/2 alleles** | 74/25/1 | 18/52/30*** | 26/38/36*** | **<0.001** | 80/18/2 | 34/52/14*** | 26/56/18*** | **<0.001** |
| **Global CDR, Mean (SD)** | 0.02 (0.10) | 0.50 (0.00)*** | 0.87 (0.33)*** | **<0.001** | 0.01 (0.07) | 0.50 (0.16)*** | 0.84 (0.36)*** | **<0.001** |
| **MMSE,**  **Mean (SD)** | 29.1 (1.1) | 27.8 (1.8)*** | 22.5 (3.3)*** | **<0.001** | 28.8 (1.4) | 27.4 (1.9)*** | 23.0 (2.8)*** | **<0.001** |
| **Global Cortical [^18^F]-Florbetapir SUVR,**  **Mean (SD)** | 1.04 (0.06) | 1.38 (0.18)*** | 1.46 (0.13)*** | **<0.001** | 1.01 (0.06) | 1.41 (0.16)*** | 1.45 (0.16)*** | **<0.001** |
| **Tau Scans, N** | 55 | 10 | 7 |  | 71 | 53 | 27 |  |

**Additional table 1.**

***MCI and DEM significantly different at p<0.05**

****MCI and DEM significantly different at p<0.01**

*****MCI and DEM significantly different at p<0.001**
