# Supplementary material for: Neurodegenerative changes in early- and late-onset cognitive impairment with and without brain amyloidosis
Source: Alzheimers Res Ther. 2020 Aug 5;12:93. doi: 10.1186/s13195-020-00647-w (PMC7409508; doi:10.1186/s13195-020-00647-w)
Supplement: Supplementary file 2 — Additional Table 2. Regional amyloid PET (18F-Florbetapir) comparisons between amyloid positive subjects for frontal, cingulate, parietal and temporal cortices. [file 13195_2020_647_MOESM2_ESM.docx]

| **Amyloid Regions** | **EOAD_MCI_** | **EOAD_DEM_** | **LOAD_MCI_­** | **LOAD_DEM_** | **ANOVA p-values** |
| --- | --- | --- | --- | --- | --- |
| **Frontal SUVR** | 1.38 (0.18) | 1.47 (0.17) | 1.41 (0.17) | 1.43 (0.17) | 0.083 |
| **Cingulate SUVR** | 1.47 (0.19) | 1.55 (0.15) | 1.50 (0.18) | 1.53 (0.18) | 0.112 |
| **Parietal SUVR** | 1.38 (0.19) | 1.47 (0.15) | 1.40 (0.17) | 1.44 (0.17) | **0.044** |
| **Temporal SUVR** | 1.27 (0.18)***** | 1.37 (0.17) | 1.30 (0.16) | 1.33 (0.16) | **0.023** |

**Additional table 2.**

***Significantly different than EOAD_DEM_ at p<0.05.**

| Amyloid Regions | EOnonAD_MCI_ | EOnonAD_DEM_ | LOnonAD­ | LOnonAD_DEM_ | ANOVA p-values |
| --- | --- | --- | --- | --- | --- |
| Frontal SUVR |  |  |  |  |  |
| Cingulate SUVR |  |  |  |  |  |
| Parietal SUVR |  |  |  |  |  |
| Temporal SUVR |  |  |  |  |  |
