# Supplementary material for: Neurodegenerative changes in early- and late-onset cognitive impairment with and without brain amyloidosis
Source: Alzheimers Res Ther. 2020 Aug 5;12:93. doi: 10.1186/s13195-020-00647-w (PMC7409508; doi:10.1186/s13195-020-00647-w)
Supplement: Supplementary file 3 — Additional Table 3. EOnonAD and LOnonAD demographic comparisons to the young and old CN groups, resp. The comparisons were done using ANOVA and chi-square tests with two-sided p-values. The Bonferroni-corrected pairwise differences relative to CN are discussed in the Results section. Significant p-values (< 0.05) are bolded. [file 13195_2020_647_MOESM3_ESM.docx]

| **Variable** | **Young CN**  **(N=145)** | **EOnonAD_MCI_ (N=113)** | **EOnonAD_DEM_**  **(N=8)** | **p-value** | **Old CN**  **(N=146)** | **LOnonAD_MCI_**  **(N=151)** | **LOnonAD_DEM_**  **(N=24)** | **p-value** |
| --- | --- | --- | --- | --- | --- | --- | --- | --- |
| **Age, years, Mean (SD)** | 69.1 (3.3) | 65.5 (5.8) | 66.3 (5.8) | **<0.001** | 79.4 (4.3) | 77.6 (6.2) | 79.4 (5.8) | **0.010** |
| **Sex,**  **Male %** | 50.3 | 50.4 | 50.0 | 0.990 | 54.1 | 59.6* | 83.3* | **0.026** |
| **Education, years, Mean (SD)** | 16.7 (2.5) | 16.2 (2.5) | 15.6 (3.5) | 0.138 | 16.7 (2.7) | 16.3 (2.5) | 15.6 (3.0) | 0.078 |
| **% *APOE* ε4, 0/1/2 alleles** | 74/25/1 | 68/30/2 | 71/14/14 | 0.066 | 80/18/2 | 86/13/2 | 83/13/4 | 0.630 |
| **Global CDR, Mean (SD)** | 0.02 (0.10) | 0.46 (0.17)*** | 0.69 (0.26)*** | **<0.001** | 0.01 (0.07) | 0.48 (0.11)*** | 0.83 (0.24)*** | **<0.001** |
| **MMSE,**  **Mean (SD)** | 29.1 (1.1) | 28.6 (1.5)*** | 23.0 (2.4)*** | **<0.001** | 28.8 (1.4) | 28.4 (1.6)*** | 23.6 (1.9)*** | **<0.001** |
| **Global Cortical [^18^F]-Florbetapir SUVR,**  **Mean (SD)** | 1.04 (0.06) | 1.03 (0.08) | 1.04 (0.08) | 0.749 | 1.01 (0.06) | 1.01 (0.09) | 1.01 (0.10) | 0.986 |
| **Tau Scans, N** | 55 | 38 | 3 |  | 71 | 51 | 2 |  |

**Additional Table 3.**

***MCI and DEM significantly different at p<0.05**

****MCI and DEM significantly different at p<0.01**

*****MCI and DEM significantly different at p<0.001**
