# Supplementary material for: Neurodegenerative changes in early- and late-onset cognitive impairment with and without brain amyloidosis
Source: Alzheimers Res Ther. 2020 Aug 5;12:93. doi: 10.1186/s13195-020-00647-w (PMC7409508; doi:10.1186/s13195-020-00647-w)
Supplement: Supplementary file 4 — Additional Table 4. Regional amyloid PET (18F-Florbetapir) comparisons between amyloid negative subjects for frontal, cingulate, parietal and temporal cortices. [file 13195_2020_647_MOESM4_ESM.docx]

| **Amyloid Regions** | **CN** | **EOnonAD_MCI_** | **EOnonAD_DEM_** | **LOnonAD­_MCI_** | **LOnonAD_DEM_** | **ANOVA p-values** |
| --- | --- | --- | --- | --- | --- | --- |
| **Frontal SUVR** | 1.01 (0.07) | 1.01 (0.06) | 1.05 (0.08) | 1.01 (0.08) | 1.06 (0.22) | 0.304 |
| **Cingulate SUVR** | 1.11 (0.08) | 1.11 (0.09) | 1.14 (0.10) | 1.10 (0.09) | 1.15 (0.22) | 0.187 |
| **Parietal SUVR** | 1.02* (0.07) | 1.02 (0.07)* | 1.06 (0.06) | 1.02 (0.08)* | 1.09 (0.24) | **0.0063** |
| **Temporal SUVR** | 0.96 (0.06) | 0.95 (0.06) | 0.98 (0.03) | 0.95 (0.07) | 1.01 (0.25) | 0.112 |

**Additional Table 4.**

***Significantly different than LOnonAD_DEM_ at p<0.05.**
