# Supplementary figures and images for: Neurodegenerative changes in early- and late-onset cognitive impairment with and without brain amyloidosis
Source: Alzheimers Res Ther. 2020 Aug 5;12:93. doi: 10.1186/s13195-020-00647-w (PMC7409508; doi:10.1186/s13195-020-00647-w)

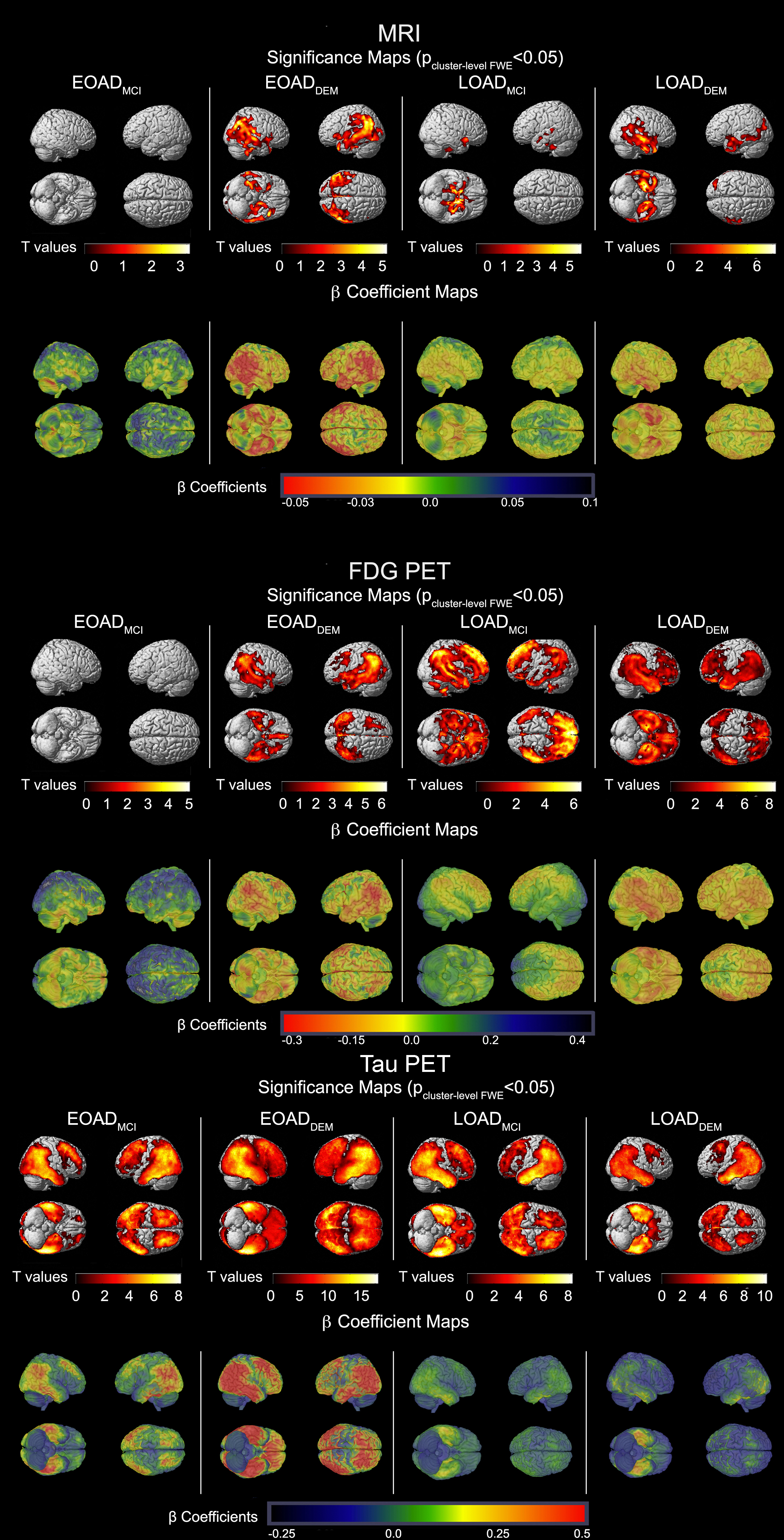

Supplement: Supplementary file 5 — Additional Figure 1. MRI (top), FDG PET (middle), tau PET (bottom) comparisons between the AD and CN groups restricted to only subjects with available tau PET scans. The significance maps show p < 0.05 thresholded FWE cluster-level corrected results. of EOADMCI (N = 10), EOADDEM (N = 7), LOADMCI (N = 53) and LOADDEM (N = 27) vs. CN (N = 126). [file 13195_2020_647_MOESM5_ESM.png]

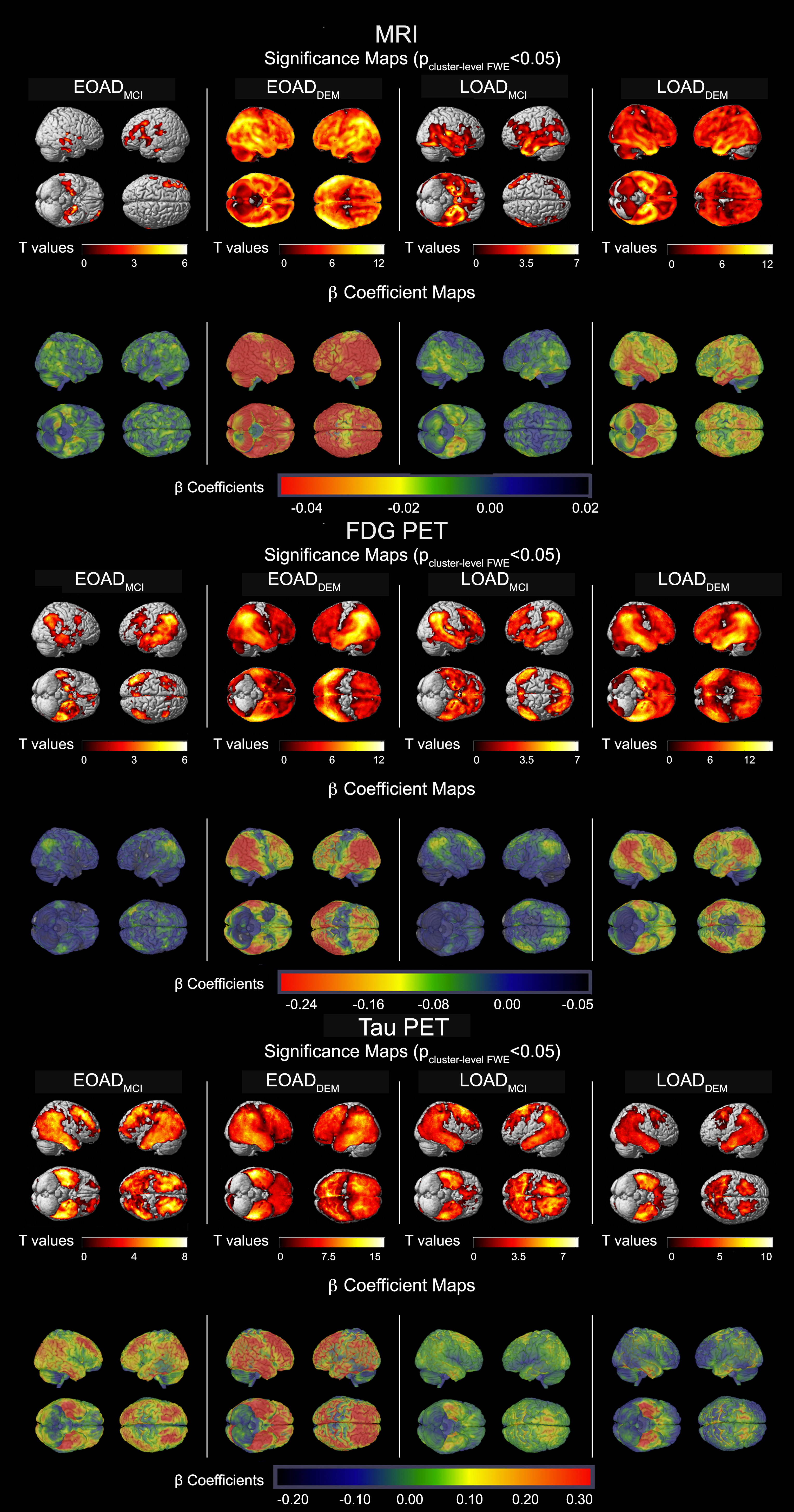

Supplement: Supplementary file 6 — Additional Figure 2. MRI (top), FDG PET (middle), tau PET (bottom) comparisons between young CN and EOAD and old CN and LOAD groups. The significance maps show p < 0.05 thresholded FWE cluster-level corrected results of EOADMCI (N = 60) and EOADDEM (N = 50) vs young CN (N = 145), LOADMCI (N = 216) and LOADDEM (N = 148) vs. old CN (N = 146). [file 13195_2020_647_MOESM6_ESM.png]

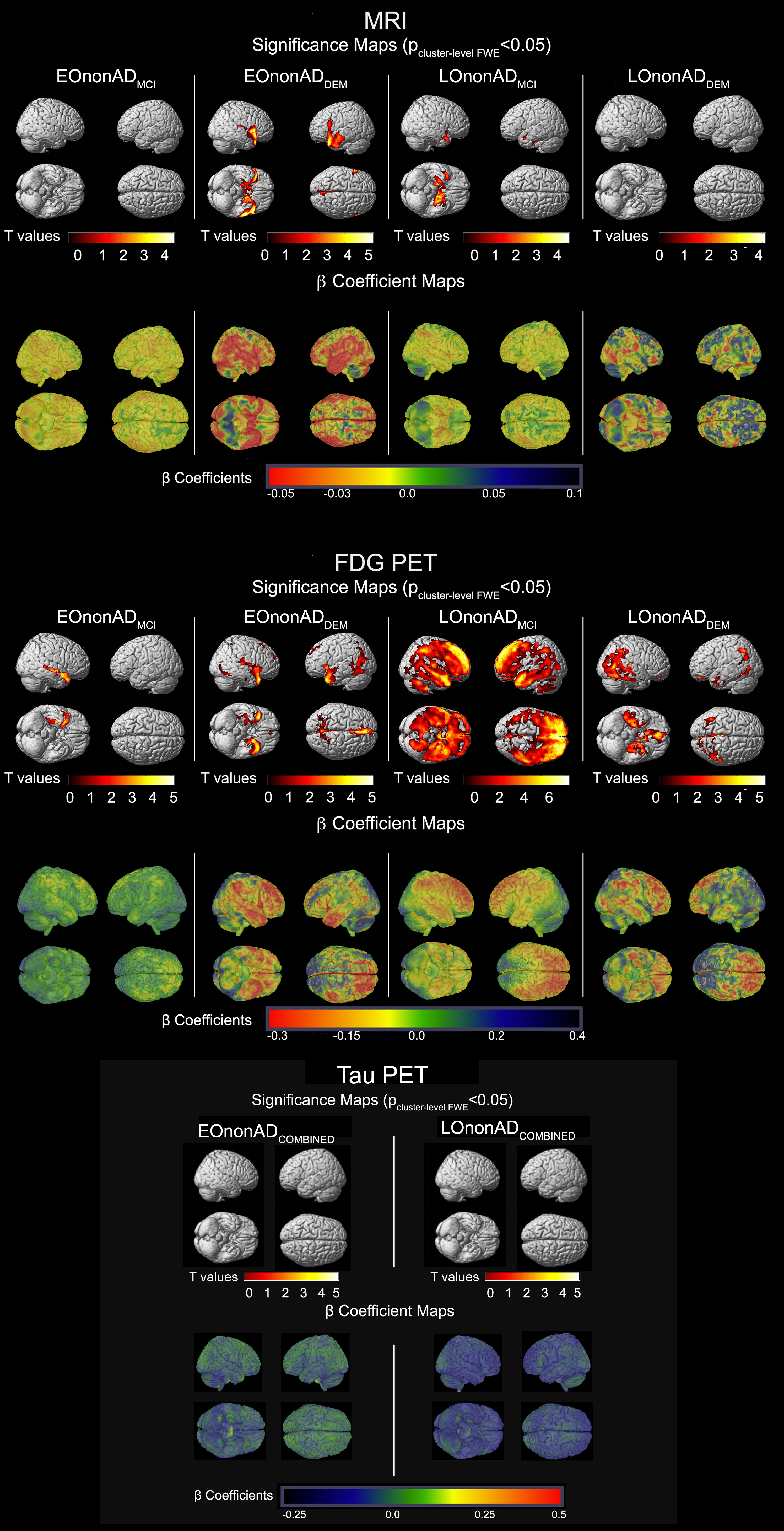

Supplement: Supplementary file 7 — Additional Figure 3. MRI (top), FDG PET (middle), tau PET (bottom) comparisons between the nonAD and CN groups restricted to only subjects with available tau PET scans. The significance maps show p < 0.05 thresholded FWE cluster-level corrected results of EOnonADMCI (N = 38), EOnonADDEM (N = 3), LOnonADMCI (N = 51) and LOnonADDEM (N = 2) vs. CN (N = 126). [file 13195_2020_647_MOESM7_ESM.png]

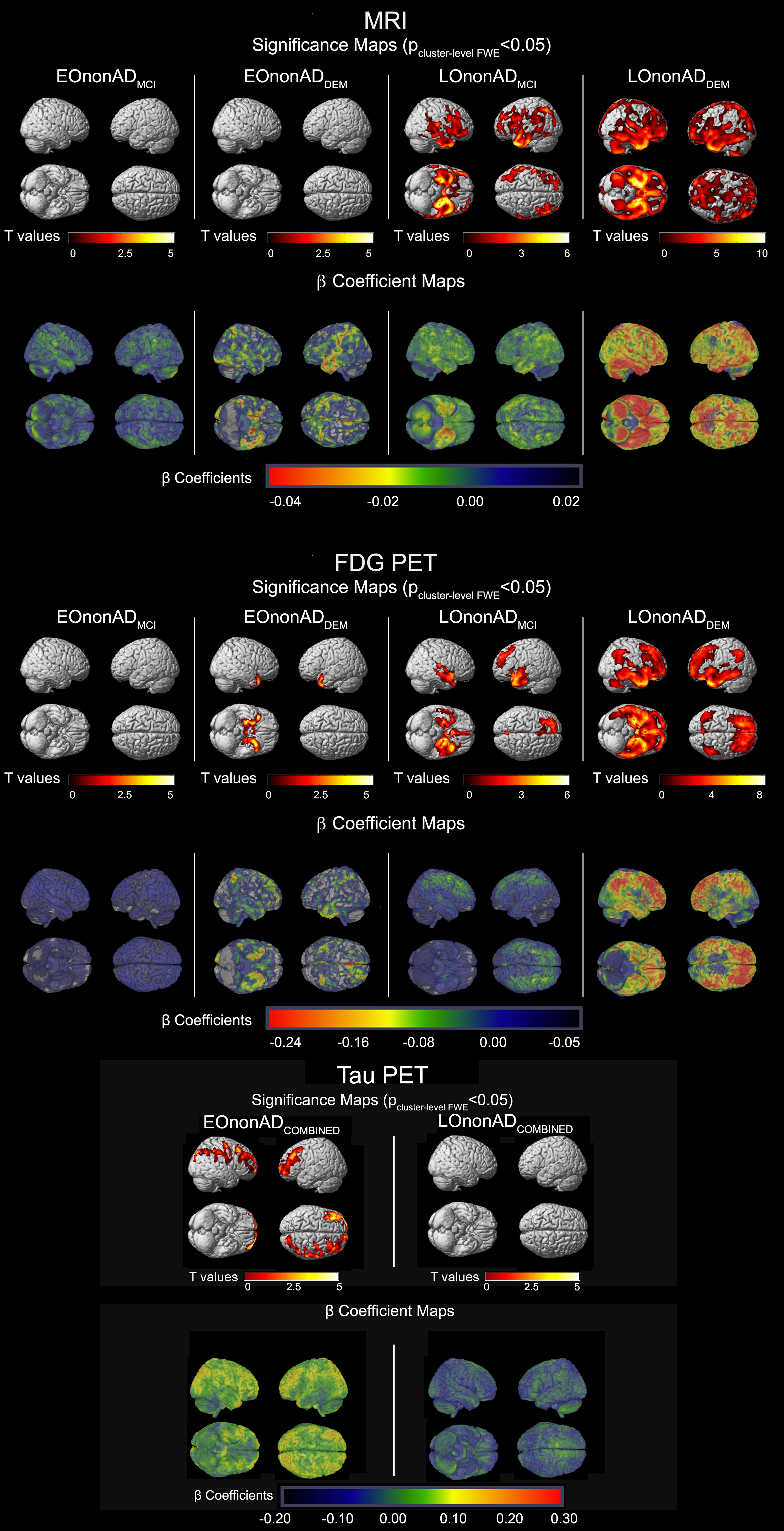

Supplement: Supplementary file 8 — Additional Figure 4. MRI (top), FDG PET (middle), tau PET (bottom) comparisons between young CN and EOnonAD and old CN and LOnonAD groups. The significance maps show p < 0.05 thresholded FWE cluster-level corrected results of EOnonADMCI (N = 113) and EOnonADDEM (N = 8) vs young CN (N = 145), LOnonADMCI (N = 151) and LOnonADDEM (N = 24) vs. old CN (N = 146). [file 13195_2020_647_MOESM8_ESM.png]
